# Presence of coronaviruses in the common pipistrelle (*P*. *pipistrellus*) and Nathusius´ pipistrelle (*P*. *nathusii*) in relation to landscape composition

**DOI:** 10.1371/journal.pone.0293649

**Published:** 2023-11-29

**Authors:** Laura Jaramillo Ortiz, Lineke Begeman, Marcel Schillemans, Thijs Kuiken, Willem Frederik de Boer

**Affiliations:** 1 Wildlife Ecology and Conservation Group, Wageningen University and Research, Wageningen, The Netherlands; 2 Department of Viroscience, Erasmus University Medical Centre, Rotterdam, The Netherlands; 3 Dutch Mammal Society (Zoogdiervereniging), Nijmegen, The Netherlands; University of Oklahoma Norman Campus: The University of Oklahoma, UNITED STATES

## Abstract

Changes in land use can modify habitat and roosting behaviour of bats, and therefore the transmission dynamics of viruses. Within bat roosts the density and contact rate among individuals increase and may facilitate the transmission of bat coronaviruses (CoVs). Landscape components supporting larger bat populations may thus lead to higher CoVs prevalence, as the number of roosts and/or roost size are likely to be higher. Hence, relationships between landscape composition and the presence of CoVs are expected to exist. To increase our understanding of the spread and shedding of coronaviruses in bat populations we studied the relationships between landscape composition and CoVs prevalence in the species *Pipistrellus pipistrellus* and *Pipistrellus nathusii*. Faecal samples were collected across The Netherlands, and were screened to detect the presence of CoV RNA. Coordinates were recorded for all faecal samples, so that landscape attributes could be quantified. Using a backward selection procedure on the basis of AIC, the landscape variables that best explained the presence of CoVs were selected in the final model. Results suggested that relationships between landscape composition and CoVs were likely associated with optimal foraging opportunities in both species, e.g. nearby water in *P*. *nathusii* or in areas with more grassland situated far away from forests for *P*. *pipistrellus*. Surprisingly, we found no positive association between built-up cover (where roosts are frequently found) and the presence of bat-CoVs for both species. We also show that samples collected from large bat roosts, such as maternity colonies, substantially increased the probability of finding CoVs in *P*. *pipistrellus*. Interestingly, while maternity colonies of *P*. *nathusii* are rarely present in The Netherlands, CoVs prevalence was similar in both species, suggesting that other mechanisms besides roost size, participate in the transmission of bat-CoVs. We encourage further studies to quantify bat roosts and colony networks over the different landscape compositions to better understand the ecological mechanisms involved in the transmission of bat-CoVs.

## Introduction

Bats are potential hosts of a wide range of viruses as a result of the high species diversity [1, 2]. More than 30 different species of Coronaviruses (CoVs) have been identified in bats from all over the world [2], including different bat species from Europe [3–6]. Severe Acute Respiratory Syndrome (SARS) virus and SARS-CoV-2 are examples of viruses for which bat-CoVs have been identified as ancestors [7–9], however there is little information regarding CoV shedding patterns and infection dynamics in bat populations [1, 4, 10]. Land use change has intensively modified the habitat and behaviour of bats [7, 11], and in consequence also the spatial characteristics of communal roosting, which may affect transmission dynamics of viruses [10, 12]. This lack of knowledge limits control strategies of potential spillover events and urges a better understanding of the spatial dynamics of bat-CoVs [10, 12–14].

Landscape composition can impact the size of bat populations in human-inhabited areas by influencing the size and number of roosts, and by changing foraging activity patterns [7, 15]. Thus, changes in landscape composition, due to e.g. deforestation, urbanization or agriculture intensification, may increase zoonotic disease risk by facilitating contact rates and possible spillover events from bats to other host species, including humans [10, 11, 16]. For instance, in Australia Hendra virus spillover has been associated with changes in bat roosting behaviour and increased contact rates with horses as a result of habitat loss and nutritional stress [11], triggering spillover events from horses to humans [17–19]. Similarly, studies in Malaysia found that forest fragmentation and agricultural intensification were associated with spillover events of Nipah virus [7]. In general changes in landscape can affect the epidemiology of pathogens, such as shown for fruit bat species (*Pteropus* spp.) [12]. Likewise, landscape structure is expected to be important in insectivorous bats such as the common pipistrelle (*Pipistrellus pipistrellus*) and the Nathusius´ pipistrelle (*Pipistrellus nathusii)*, since they also depend on existing landscape resources for roosting and hunting, and are common reservoirs of coronaviruses [4, 20].

Physical contact among bats determines the hosts’ contact network and thereby pathogen transmission probability [21]. As gregarious animals, many bat species aggregate in different types of colonies depending on the season and intrinsic characteristics (i.e. sex, age). Contact among bats mainly occurs while roosting, establishing social networks for grooming, nursing, mating, hibernating and other social behaviours [21, 22]. In The Netherlands, large roosts (i.e. maternity colonies) of the common pipistrelle can hold 40 to 400 individuals [23]. In contrast, *P*. *nathusii* is a migratory species and maternity colonies are usually found in North-Eastern Europe, while mating roosts (containing few individuals) are present in The Netherlands [24, 25]. Within roosts the spread of bat-CoVs may be facilitated as contact rates are higher and social networks are denser with more and stronger connections among individuals [4, 21, 26]. Furthermore, studies performed on European bat species found that juvenile bats are more susceptible to bat-CoVs, since these naïve individuals have not yet developed immunity [4, 27]. Hence, juvenile bats are likely to enable pulses of viral transmission in maternity colonies before they become immune [4]. Due to higher contact rates and the presence of susceptible individuals, maternity colonies can be important amplifiers of bat-CoVs [4, 21].

CoV transmission can also be influenced by the number of roosts in an area and the contact network within and between these roosts [26], which occurs at different spatial scales in *P*. *pipistrellus* and *P*. *nathusii*. During mating season, females of both species leave their maternity colonies and form new and smaller mating groups with adult males [23]. Mating roosts of *P*. *pipistrellus* are frequently found at a short distance from the maternity colony [23]. In contrast, mating roosts of *P*. *nathusii* are present along migratory routes [24, 25]. Viral transmission among bat roosts can occur from local spreading to migratory movements connecting different roosts over larger distances. At both scales, landscape composition indirectly influences transmission dynamics between hosts and viruses through its effects on bat densities, dispersal, and contact networks [7, 20].

Few studies have investigated the ecological associations between landscape composition and the presence/size of bat roosts in The Netherlands. In Northern-Europe, studies found that colonies from *P*. *pipistrellus* and *P*. *nathusii* were more likely located near suitable foraging sites, where roosting opportunities are higher (i.e. structure availability) and resources are abundant [28–30]. Habitats with high resource abundance can increase bat population density by favouring birth rates, survival, and range expansion [7, 31]. In terms of roosting opportunities, both natural (i.e. large trees, caves, burrows) and human-made structures (i.e. buildings, bridges) can be occupied by bats for roosting [21, 32, 33]. Buildings (i.e. churches, domestic houses, barns) in particular are frequently occupied by large groups of *P*. *pipistrellus* in The Netherlands (*i*.*e*. maternity colonies), offering suitable microclimatic conditions and hiding possibilities [23].

As landscape composition can influence bat population size (i.e. roost size, number of roosts), and thereby bat densities and contact rates [21, 30, 34], landscape variables may influence CoV transmission [7, 20]. Therefore, under the assumption that landscape composition influences the density of bats [7, 20], the relationships among landscape variables and virus presence were investigated. Hence to better understand the ecological and spatial transmission dynamics of CoVs in European bat species, this study aims to analyse how landscape variables are related to the presence of bat-CoVs in The Netherlands. We hypothesised that suitable landscapes for roosting and hunting opportunities, which differ among these bat species, will support a higher density of bats and thus, will increase the probability of finding bat-CoVs [4, 7, 26].

## Materials and methods

### Bat species

The common pipistrelle (*P*. *pipistrellus*) and the Nathusius`pipistrelle (*P*. *nathusii*) are edge-foragers and the most widespread bat species in The Netherlands [35–37]. Studies have identified the main activity of *P*. *pipistrellus* within a home range of 2 km radius during mating and maternity seasons [29], while in *P*. *nathusii*, the home range has been reported within 6.6 km radius [30, 38]. As a migratory species, *P*. *nathusii* can cover up to 1900 km [38]. The roost location and foraging activity (28–30)of bats are influenced by differences in habitat quality [35, 38].

### Sample collection

Faecal samples from *P*. *pipistrellus* and *P*. *nathusii* were collected throughout The Netherlands from 2017 to 2019 (S1 Fig), by volunteers guided by the Dutch Mammal Society, in collaboration with the Erasmus University Medical Centre (EMC), within the framework of Zoonoses in the Night project (ZITN, grant agreement no. 522003002”). Faecal samples were collected from bat boxes, buildings, caves and also from bats in wildlife rehabilitation centres. Sampling was performed in spring, summer, autumn, and part of the winter. However, as few samples were collected during spring and winter, we only considered samples collected in summer and autumn for the analysis, covering maternity and mating season in *P*. *pipistrellus*, and migratory/mating season in *P*. *nathusii* (S2A and S2B Fig). Samples from wildlife rehabilitation centres were only included in the analysis if the original location of the individual was known (i.e. the exact coordinates where the animal was originally found). For each faecal tube that was collected, the geographic coordinates and the species was recorded. A sample unit corresponded to one faecal tube collected from a single bat or from a single faecal pile within a roost. Volunteers were asked to put up to five faecal pellets (if available) from the same pile on each tube, so that one tube contained a maximum of five faecal pellets (*i*.*e* 88% of all tubes had 5 pellets in *P*. *Pipistrellus*, and 73% in *P*. *nathusii*). Varying per sampling site, 1 to 10 tubes with faeces were collected, and each tube was always tested separately for the presence of bat-CoVs. Bat species and roost type (*i*.*e*. small/large roost) were determined by trained volunteers coordinated by the Dutch Mammal Society. Species identification was based on visual inspection of the bat, sounds recorded at the site of collection, and previous knowledge of the roosts. Collected tubes had a volume of 2 mL and contained 1 mL of virus transport medium [39]. Faecal samples were stored at 4°C and within a maximum of 11 days after collection (S3 Fig), samples were transported to the research facility, and stored at -80°C before being screened for RNA of bat-CoVs. No effect between the number of days at transport medium and the presence of CoVs was observed.

### Coronavirus RNA detection

Each tube with faeces was tested for the presence of coronavirus RNA. Tubes were thawed, virus transport medium and faecal pellets were mixed by using the back of a wooden swab to resuspend the pellet, and subsequently vortexed for 15 seconds. An approximate volume of 200 μl of this faeces–virus transport medium mixture was then added to a tube containing 900 μl S.T.A.R. buffer (Roche) and 120 μl chloroform. The mixture was again vortexed for 15 seconds, then centrifuged for 5 minutes at 1000 rpm. Supernatant (200 μl) was added to 300 μl lysis buffer (MagNA Pure external) and RNA was extracted mechanically (MagNA Pure LC Total Nucleic Acid Isolation Kit). cDNA was synthesised using SuperScript IV First-Strand cDNA Synthesis Reaction (ThermoFisher) according to the manufacturer’s instruction. A nested PCR protocol for the detection of coronaviruses was then used [40]. Products were loaded on gel, and bands of the right size (approximately 474 base pairs) were cut out of the gel, purified using a QIAquick Gel Extraction Kit (Qiagen) in accordance with the manufacturer’s instructions and amplified for sequencing. PCR products were sequenced using backward (5’ CTTATGGGTTGGGATTATCCTAAGTGTGA 3’ and 5’ CTTATGGGTTGGGATTATCCCAAATGTGA 3’) and reverse primers (5’ CACACAACACCTTCATCAGATAGAATCATCA 3’ [40]; using a Big Dye Terminator Cycle v3.1 kit in accordance with the manufacturer’s instructions in an Applied Biosystems Hitachi 3130xl Genetic Analyzer to check if the PCR product was indeed from a coronavirus. Lastly, RdRp fragments of 395 to 396 nt in length were obtained from PCR positive samples.

### Landscape predictors

The landscape variables were obtained from three main data sources: (1)- LGN7 (National land-use grid file, The Netherlands, version 7), which contains raster data of land use cover with a spatial resolution of 25*25 m. (2)- Boombasis Data (Tree Base Data), obtained from the Boomregister Data (Tree Register). The last database contains the height of trees based on AHN2 and AHN3 raster data (i.e. object height, The Netherlands). Lastly, we used (3)- BAG-2018 Database (i.e. the Basic Registration of Buildings and Addresses), which contains polygon data of the age and location of buildings [41].

Using ArcGIS-Pro version 2.5, landscape attributes were obtained for each faecal sample in several radius sizes, which are defined as buffers (S1 Table in S1 File). These buffers were intended to cover the different spatial scales within the home range of each bat species, so that we could identify the best-fitting buffer size at which landscape variables and elements were selected [36].

In accordance with the ecological characteristics of bats for roosting and foraging activity [29, 38, 42–45], a total of 16 landscape variables were initially extracted from ArcGIS for the spatial analysis. Land use subclasses from the LGN7 data base were grouped into nine categories (S2 and S3 Tables in S1 File). For each buffer, the percentage of the area (m^2^) covered by each land use type was calculated. We considered the mean/min distance to fresh water and forest as basic ecological resources for hydration and foraging activity [29, 42]. Since old buildings may facilitate the accessibility for bats (i.e. more wall cavities, hiding spaces available, no isolation wall) and support different colony sizes [46, 47], we added a building variables representing the cover (in percentage) of old buildings (built before 1939). For the variables distance to tree ≥10 (m) and tree cover ≥10 (%), we used a threshold of ≥10m height, as a reference of suitable trees for bats to roost [42]. In contrast, variables referring to forest cover or mean/min distance to forest included any type of trees.

### Statistical analysis

The statistical analysis was performed in R-studio, version 2022.07.0–548. First, we used logistic regression models (package lme4; [48]) for every landscape variable in four different buffer sizes to select the best fitting spatial scale for each of the landscape variables (i.e. best buffer size). For this step, all models were fitted separately, containing the presence/absence of bat-CoVs as the response variable and each landscape component (individually) at a specific buffer size as an explanatory variable. All predictor variables were standardized (mean = 0; standard deviation = 1).

Next, all landscape variables (at their best fitting buffer size) were then added as predictor variables in a multivariable logistic regression model (GzLMM, family = binomial) with CoVs presence as the response variable and location as a random factor to account for repeated sample tubes collected per location. We considered each faecal tube as our sample unit, hence repeated tubes per location (which usually differed in the presence/absence of CoVs) were considered as pseudo-replicates in the data set. To avoid multicollinearity between explanatory landscape variables [49], we used the correlation coefficient (r) with a conservative threshold of >0.4 to avoid overfitting the model. Based on this threshold, all correlated landscape variables that were not directly associated with roosting structures or habitat preferences were not included in the initial multivariable model (see S1 Appendix, e.g. Mean tree height was removed as it was correlated to Tree cover). To correct for differences in the number of faecal pellets per sample tube, the number of pellets was included as an additional explanatory variable in the multivariable logistic regression model Likewise, to account for differences in sample source, we included a nominal variable with the following sample source classes: non roosting bats (i.e. samples collected from individual bats in wildlife rehabilitation centres), roosts (i.e. samples collected from bat boxes, caves, buildings and other human-made structures) and unknown (information of the sample source was not provided by the volunteer). To consider the potential influence of sample collection seasonality on the presence of bat-CoVs we included “season” as an additional variable in the multivariable model. Once the initial multivariable logistic regression model was formulated, we followed a backward selection approach, using a Likelihood ratio test and Akaike information criterion (Δ AIC ≥ 2) as measures to compare the nested models. A detailed description of variables and model selection steps are described in the supplementary information (S1 Appendix). The resulting final model contained the landscape variables that best predicted the presence of bat-CoVs for a specific bat species. We calculated confidence intervals for all predictor variables using the Wald method, and to validate the goodness of fit of the models and evaluate the underlying assumptions, residuals and diagnostic plots were calculated using the DHARMa package. To assess the predictive capacity of the final GzLMMs (model fit) we obtained the conditional and marginal R^2^ (i.e. the proportion of variance in CoVs presence explained by the fixed effects with/without the effect of the random factors) using the performance package [50]. To calculate the contribution and effect size of each explanatory variable in relation to the variance explained in the model we use the inclusive R^2^ (package partR2 [51]) and standardized odd ratios (package effectsize [52]). We tested for spatial autocorrelation using Moran’s I test from the DHARMa package [53].

## Results

A total of 391 faecal tubes were screened for bat-CoVs in *P*. *pipistrellus* and 139 in *P*. *nathusii*, showing a similar proportion of positive samples in both bat species (0.37 in *P*. *pipistrellus* and 0.33 in *P*. *nathusii*). Phylogenetic analyses and RdRp sequence similarity suggested circulation of two distinct alpha coronaviruses in *P*. *pipistrellus* (sequences were similar to previously described: accession no. KT345296.1 and KT345294.1 [54]) and one alpha (sequence similar to accession no. OQ405400) and one beta coronavirus in *P*. *nathusii* (sequence similar to accession no. OQ405399). The sequences of the two alpha coronaviruses in *P*. *pipistrellus* showed a similarity of 94.9 to 100%, and 95.9 to 100% for each of the two alpha coronaviruses. The sequence similarity between them was lower, namely 71.9–75.7%. The sequences of the alpha coronavirus in *P*. *nathusii* showed a similarity of 98.5 to 100%. The sequences of the beta coronavirus clustered with previously detected Merbecoviruses in *P*. *nathusii*, with a sequence similarity of ≥96.7% [55].

**Fig 1:**
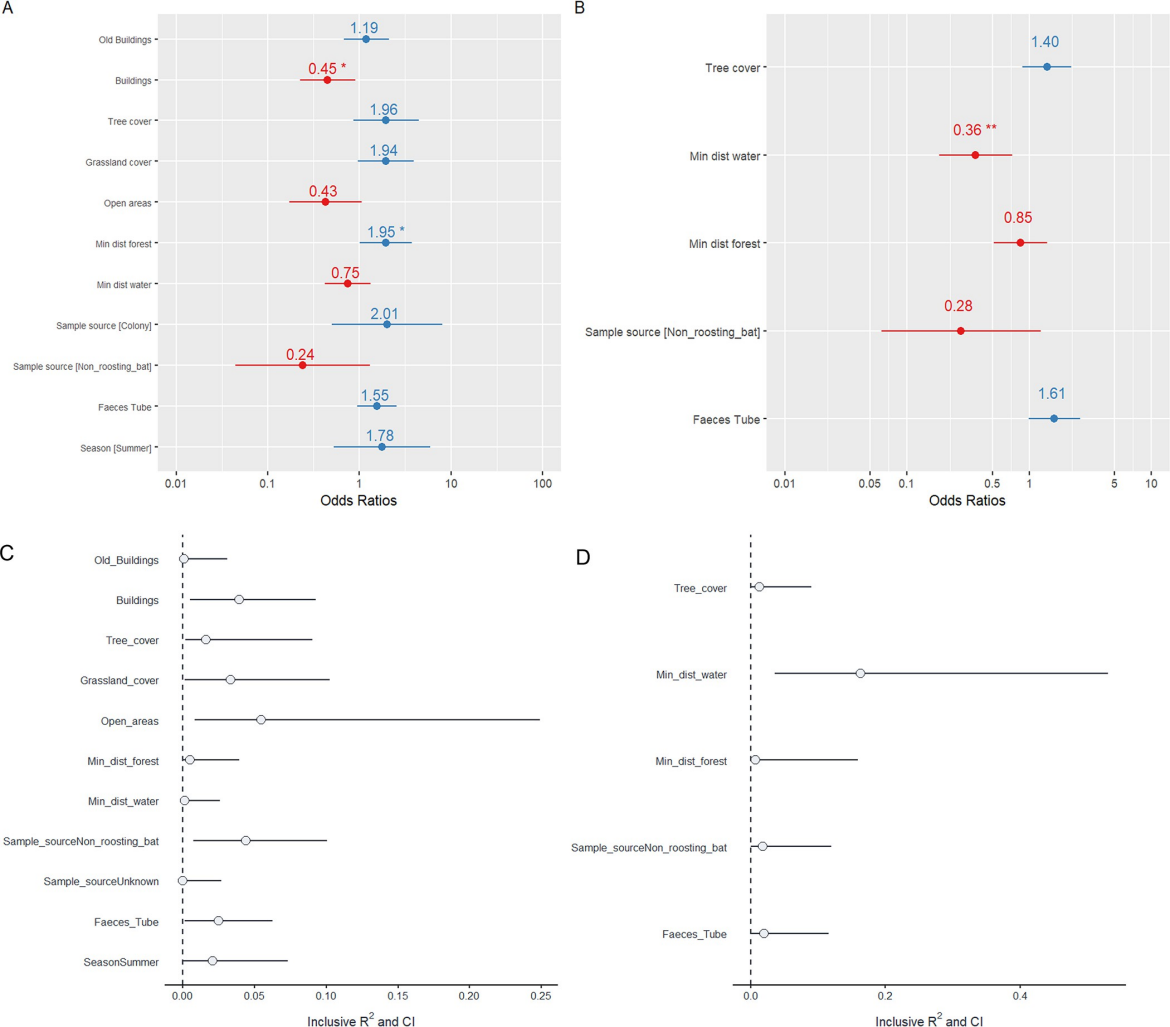
Partial coefficients and odd ratios of all predictor variables in the final model for the species *P*. *pipistrellus* (A, C) and *P*. *nathusii* (B, D). The figures above show the proportion of variance explained by each predictor variable in the final model, while accounting for the effects of other predictors (inclusive R^2^). The figures below display the relative odd ratios of the estimates, with positive relationships represented in blue and negative relationships represented in red. Values located near 1 on the x-axis indicate smaller effect sizes. Note that values are represented with their corresponding confidence intervals, and are standardized using a mean = 0; standard deviation = 1. For the variable sample source, “roost” is the reference. A detailed description of the inclusive R^2^ and odd ratios’ values can be found in S4 and S5 Tables in S1 File.

In *P*. *pipistrellus*, the selected model that best explained the presence of coronaviruses included seven landscape variables, and three additional correcting variables (Table 1 and Fig 1), namely sample source, season and the number of faecal pellets per tube. The effects of seasonality did not contribute significantly to the model (LRT, and AIC barely changed with/without seasonality), however, considering the ecological and epidemiological importance of this variable, we decided to keep seasonality in the model. Other variables such as low vegetation, infrastructure and mean tree height were not included in the initial backward selection model as these were highly collinear to other variables of larger interest (e.g. building cover, tree cover, see S1 Appendix). In the final model, the landscape attributes minimum distance to forest, grassland cover, old buildings cover and tree cover ≥10m were all positively correlated with the presence of CoVs, while building cover, distance to water, and open areas showed a negative correlation with the presence of CoVs (Fig 1 and S4A–S4G Fig). The variables with the strongest associations, in terms of effect size, were building cover and minimum distance to forests, while the relative variance (inclusive R^2^) explained by the latter was lower compared to other landscape variables, such as open areas or grassland cover (Table 1 and Fig 1).

**Table 1:** Estimates and P-values of the final GzLMMs. Correlation coefficients (β), standard errors (SE), 95% confidence intervals (CI), and p-values of all landscape variables present in the final GzLMMs for *P*. *pipistrellus* and *P*. *nathusii*. Variables representing the number of faecal pellets per tube, and sample source (reference = roosts) were included in the model as correcting variables. The term Buffer refers to the spatial size used for each predictor variable; R^2^ values correspond to the conditional and marginal proportion of variation explained by the model.

Buffer (km)	Variables	Estimates (β)	SE	CI: 2.5% 97.5%	P-value
**Landscape model Common pipistrelle (*Pipistrellus pipistrellus)***					
R^2^cond = 0.78, R^2^marg = 0.25					
	Intercept	-1.266	0.463	-2.591 0.059	0.061 .
2	Building cover	-0.799	0.356	-1.496–0.102	0.0246 *
2	Buildings age >1939	0.176	0.292	-0.395 0.747	0.546
2	Tree cover (≥10m)	0.672	0.417	-0.146 1.489	0.107
2	Min. distance forest	0.668	0.335	0.012 1.324	0.045 *
2	Grassland	0.662	0.361	0.044 1.368	0.066 .
2	Open areas	-0.853	0.467	-1.768 0.062	0.068 .
2	Min distance water	-0.289	0.295	-0.865 0.288	0.327
	Sample origin (ref = roost)	-2.124	0.803	-3.698–0.549	0.008 **
	Non roosting bat				
	Unknown	-0.699	0.711	-2.093 0.695	0.326
	Faecal pellets	0.439	0.254	-0.059 0.937	0.085 .
	Season	0.575	0.619	-0.638 1.789	0.353
**Landscape model Nathusius´ pipistrelle (*Pipistrellus nathusii)***					
R^2^cond = 0.35, R^2^marg = 0.29					
	Intercept	-0.752	0.262	-1.267–0.238	0.004 **
2	Min. distance water	-1.010	0.352	-1.699–0.321	0.004 **
3	Tree cover (≥10m)	0.339	0.235	-0.121 0.799	0.148
2	Min. distance forest	-0.163	0.256	-0.665 0.339	0.524
	Sample source (ref = roost)	-1.283	0.766	-2.785 0.219	0.094 .
	Non roosting bat				
	Faecal pellets	0.478	0.245	0.002 0.958	0.051 .

In *P*. *nathusii*, the small sample size limited the number of landscape variables to include in the initial multivariable model, given the high collinearity (e.g. infrastructure cover, open areas, mean tree height) between landscape predictors and the convergence warnings (S1 Appendix). In the end, minimum distance to water, minimum distance to forest, and tree cover were the only landscape variables retained in the final model, while sample source and faecal pellets per tube remained as correcting variables (Fig 1 and S4H Fig). In *P*. *nathusii* the variable season contributed poorly to the model and was confounding with sampling source (i.e. samples collected in the autumn were associated with higher numbers of samples collected from roosts), thus season was removed from the model. The final model showed a significant negative correlation between distance to water and the presence of CoVs (i.e. samples further away from water were less likely to carry CoVs). Moran’s I test indicated no significant spatial autocorrelation in the model residuals of *P*. *nathusii* (p = 0.098) and *P*. *pipistrellus* (p = 0.354), justifying the decision to not include distances between samples in the model as an additional covariate.

**Fig 2:**
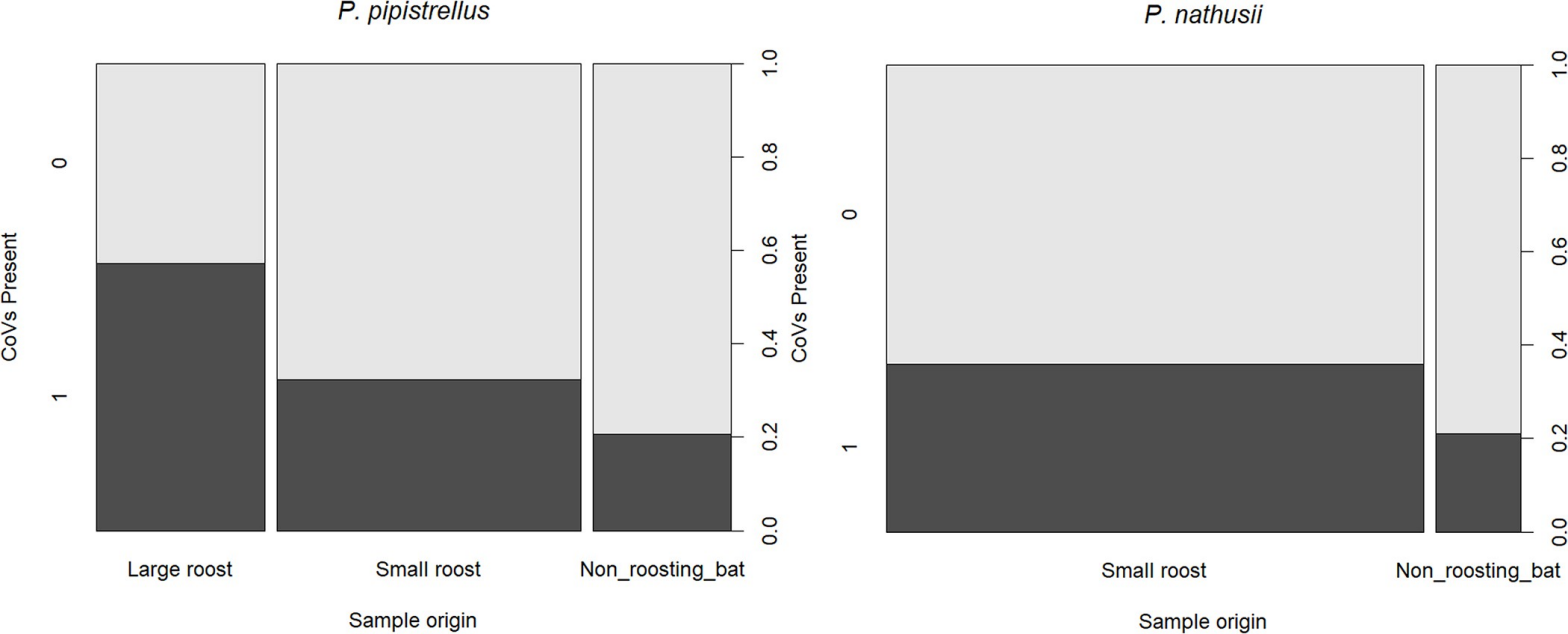
Presence of CoVs in faecal samples collected from non-roosting bats, small roosts and large roosts. On the y-axes, 0 = absence of CoVs (light grey) and 1 = presence of CoVs (dark grey). The proportion of positives/negatives is given per sample source and is represented on a scale from 0 to 1. In *P*. *nathusii* no samples were available from large roosts as maternity colonies are rarely found in The Netherlands. The width of the bars is relative to the sample size of each sample source category.

From all correcting variables, the sample source explained most of the variation in *P*. *pipistrellus*. Samples collected from non-roosting bats in rehabilitation centres had a lower probability of CoVs, compared to samples collected from bat roosts. To better visualise differences in roost size and the presence of CoVs in *P*. *pipistrellus* we formulated a second GLzMM. In this model, we re-categorised samples from roosts into two groups: small roosts, consisting of bat boxes with few individuals, and large roosts, primarily composed of buildings containing tens of individuals. To address confounding effects between the variables season and roost size (large maternity colonies are present during the summer, while smaller roosts are common in autumn, see S5 Fig), we excluded season from the model. We included the same landscape predictors as in the final model, the number of faecal pellets per tube as a covariate, and location as a random factor to account for repeated sample tubes per location (same predictor variables as in the previous models). In addition, procedures for model validation and goodness of fit were applied as before (conditional R^2^ = 0.79, marginal R^2^ = 0.33). After including all these considerations, we observed that the proportion of positive samples for CoVs was different depending on the roost size (Fig 2), as large roosts had a higher number of positives in *P*. *pipistrellus* when compared to small roosts (-2.642, p<0.05) and samples collected from non-roosting animals (bats in rehabilitations: -3.36, p<0.005). No significant difference in the presence of CoVs was observed between small roosts and non-roosting bats. In the case of *P*. *nathusii*, associations between roost size and the presence of CoVs were not further analysed as samples were never collected from large roosts (large roosts are rarely present in The Netherlands [23]), and very few samples from *P*. *nathusii* were collected from non-roosting bats (S5B Fig).

## Discussion

We investigated the spatial relationships between landscape composition and CoVs presence in faecal samples of *P*. *pipistrellus* and *P*. *nathusii*. We hypothesised that landscape components supporting larger bat densities (*i*.*e*. providing structure availability for roosting and suitable conditions to forage; [7, 26, 56]) will increase the probability of finding CoVs. Results showed that locations nearby fresh water bodies had a higher probability of CoV presence in *P*. *nathusii*. For *P*. *pipistrellus*, grassland, large tree cover, and areas further away from forest, were associated with CoVs presence, while building cover and open areas presented negative correlations. The above suggests that some landscape attributes explaining the presence/absence of bat CoVs are likely associated with the species’ foraging strategies, while positive associations with cover from large trees (>10m) might be attributed to the availability of roosting sites. Additionally, the best spatial fit for the correlations between landscape variables and the presence of bat-CoVs remained within the core home range and core surroundings in both species, where presumably most of the foraging activity takes place and roosts are likely to be close [29, 38].

Landscape associations with CoVs may be explained by the proximity to suitable foraging sites that favour bat population size (e.g. higher number of roost sites) [7]. In *P*. *nathusii*, locations close to fresh water significantly increased the probability of finding CoVs. In fact, no positive samples were observed when the minimum distance to water was greater than 400m (S4H Fig). Wetlands and riparian habitats have been frequently associated with large populations of *P*. *nathusii*, providing optimal hunting opportunities and routes for migration [30, 38, 45]. Correlations with distance to water were only weakly associated for the generalist edge forager *P*. *pipistrellus*, which mostly hunts at the edge of vertical structures like trees and vegetation [29, 45, 57], including natural unimproved grasslands containing higher insect abundance and diversity [44]. Accordingly, areas further away from forest (up to 400 m) and high grassland cover were associated with a higher chance of detecting bat-CoVs. Bare open areas presented negative associations with the presence of CoVs in *P*. *pipistrellus*. These areas might lack the structural complexity that is needed to successfully forage or establish roosts [58–60].

Besides providing foraging opportunities, correlations between landscape components and the presence of CoVs can also be viewed more directly via the availability of roosting structures. We observed a positive correlation with cover of large trees (>10m, more frequently used for roosting) and the presence of CoVs in *P*. *pipistrellus* and *P*. *nathusii*, although this positive effect was not significant. Surprisingly, we found a significant negative association between building cover and the presence of CoVs, even though large roosts (*i*.*e*. maternity colonies) of *P*. *pipistrellus* are frequently found in buildings throughout the Netherlands [23]. Previous studies in England found negative associations between bat abundance and large, homogenous urban areas when green areas and structural variation were scarce (e.g. parks, water bodies) [61]. In this study, distinctions between the building types cannot be made since all building categories (i.e. primarily built-up areas, secondary built-up areas, rural buildings) were pooled in one variable (S2 Table in S1 File). Hence, to better understand the spatial relationships of these landscape variables and CoVs presence, a more detailed landscape classification in combination with the associated landscape heterogeneity (i.e. the spatial context) at different scales should be considered [36, 62, 63]. Old buildings (built before 1939) cover (e.g. churches), in which maternity roosts are often found [47, 64], remained in the final model of *P*. *pipistrellus* but showed a weak positive correlation with CoVs presence; the coarse spatial resolution, the absence of a stratified sample design, and our sample size might not be appropriate to detect such effect.

The relationships between landscape composition and bat-CoVs [7, 15] can be influenced by other factors such as those related to host and virus intrinsic characteristics (i.e. host-virus interactions). For example, landscapes containing poor resources (e.g. low insect abundance, light/chemical pollution) can negatively influence the fitness and immune response of bats due to nutritional, deficiency [7, 14] or physiological stress [7, 65, 66], making them more susceptible to CoVs [11]. Moreover, variation in landscape composition may influence microclimatic conditions (i.e. humidity, U.V. exposure) and the survival of CoVs in the environment [67]. These fine-scale environmental or host-related factors likely influence CoVs circulation and may be reflected in the models with a relatively high proportion of variance explained by the location over the main effects (high conditional R2 vs marginal R2), particularly in *P*. *pipistrellus*. Hence, possible explanations referring to correlations between CoVs and landscape composition remain speculative and need further research on different ecological fields (e.g. eco-immunology, disease ecology, physiology, virology) to better understand the underlying factors behind these correlations. Despite the lower number of parameters present in the final model of *P*. *nathusii*, conditional and marginal R^2^ did not differ considerably, suggesting that the included fixed factors were relatively more important in this model.

In our study, the proportion of faecal samples belonging to large bat roosts in *P*. *pipistrellus* had a significantly higher probability to be positive for CoVs, compared to samples collected from smaller roosts (Fig 2). The obtained results are in accordance with other studies that have identified roost size as an important factor influencing CoVs transmission in other bat species [1, 4, 26, 68]. However, a smaller quantity of faecal tubes was collected from non-roosting bats (See Fig 2), and we cannot be certain that all collected faecal tubes from roosts corresponded to independent bat individuals (e.g. same bat individual might had defecated in different faecal piles within short time), hence it is possible that some bat individuals are overrepresented in this study. Furthermore, roost size classes (small roost/large roost) were estimated by visual inspection (e.i few individuals roosting in bat boxes vs tens of individuals roosting in building structures), but the actual number of bats within a roost was not specified. In Germany, studies found that maternity colonies in particular were important amplifiers of CoV transmission in several insectivorous species. This was explained by roost size but also by the addition of susceptible new-borns added to the colony [4, 5]. Accordingly, our study shows that large roosts from *P*. *pipistrellus* have a significant higher probability to test CoV positive and due to the sampling seasonality (summer), most of these likely correspond to maternity colonies (female bats) with immunological naive individuals. Thus, colony size and new-borns’ susceptibility, may be important factors influencing bat-CoVs transmission in Dutch populations of *P*. *pipistrellus*.

Interestingly, the percentage of positive samples for CoVs was similar in *P*. *pipistrellus* and *P*. *nathusii* (33% and 37%, respectively), despite the differences in roosting behaviour and movement patterns of both species in The Netherlands. Other studies have reported a similar range of CoVs in samples of *P*. *nathusii* (9–37%) [5] and *P*. *pipistrellus* (2–22%) [3, 9]. Nevertheless, in our study sample units do not directly correspond to a bat individual, thus direct comparisons cannot be made. Despite the fact that samples of *P*. *nathusii* were not collected from maternity colonies containing new-born populations (maternity roosts are rarely present in the Netherlands), the probability of detecting CoVs was similar in both bat species. This may indicate that in *P*. *nathusii*, other mechanisms besides roost size or juvenile susceptibility are involved maintaining the circulation of bat-CoVs in The Netherlands (e.g. heterogeneities in species competence, movement patterns and colony networks during migration, [69]) and need further investigation.

We encourage further studies to investigate the underlying ecological mechanisms involved in landscape epidemiology of bat CoVs by first investigating directly how landscape compositions and land use changes influence the roosting ecology (e.g. number of roosts, roost size) and contract networks of insectivorous bats in The Netherlands, particularly in reference to maternity colonies. Moreover, due to an unbalanced sampling collection in both species (i.e. most of the sample tubes collected were in the summer for *P*. *pipistrelles* and autumn for *P*. *nathusii* (S5 Fig), seasonality did not have a significant statistical effect on the presence of CoVs. Seasonality is known to be an important component in the transmission of CoVs since roost size, social network contacts, movement patterns, and demographic composition (i.e. number of susceptible individuals) all fluctuate [4, 26, 27] depending on the season [4, 26, 27]. To capture this effects, further longitudinal studies are needed to monitor CoVs infection dynamics and contact networks across several seasons and years. Lastly, looking at species heterogeneities and individual traits (e.g. age, sex, body mass, immune responses) may also shed light on demographic and physiological factors facilitating the transmission of bat-CoVs [27].

## Conclusion

This study describes the relationships between landscape composition variables and the presence of bat-CoVs in *P*. *pipistrellus* and *P*. *nathusii*. Preliminary results suggest that some landscape attributes explaining the presence/absence of bat CoVs are likely associated with food-rich environments and foraging strategies, which differ among bat species. Correlations with roosting structure variables (*i*.*e*. large trees, building cover, old buildings) were weak or showed a negative association with the presence of CoVs. We also show that large roosts, likely from seasonal maternity colonies, had a significantly higher probability of containing CoVs in *P*. *pipistrellus*. Even though maternity colonies of *P*. *nathusii* are not present in The Netherlands, the probability of detecting CoVs was similar in both species, suggesting that other factors besides roost size participate in the transmission of bat-CoVs.

## Supporting information

S1 Fig Spatial distribution of faecal sample tubes collected for the study between 2017–2019 in the Netherlands. Source Basemap layer: World Topographic Map. Public domain Metadata. Credits attribution: Kadaster NL, Esri Inc, HERE, Garmin, FAO, NOAA, USGS. ArcGIS-Pro 2.5. https://services.arcgisonline.com/ArcGIS/rest/services/World_Topo_Map/MapServer Licensed under the Esri Master License Agreement.
(TIF)

S2 Fig Temporal distribution of the number of tubes collected between 2017–2019, and the proportion of positive samples in *P*. *pipistrellus* (A) and *P*. *nathusii* (B).
(ZIP)

S3 Fig Correlation between transportation days and the presence of CoVs in faecal samples kept on transportation medium for 1–11 days. No association is observed. (TIF)

S4 Fig Correlations between landscape variables and presence of coronaviruses in *P*. *pipistrellus* (A-G) and *P*. *nathusii* (H). On the x-axis of each figure, histograms are displayed showing the distribution of each landscape variable across all faecal samples that are positive (above) and negative (below) for CoVs. On the y-axis the probability of coronavirus detection is represented. For *P*. *pipistrelle* min. distance to water (A), building cover (B), and open areas (C) were negatively correlated with the presence of CoVs, while grassland (D), minimum distance to forest (E), tree cover (> = 10m; F) and old buildings (G) had a positive correlation. In *P*. *nathusii*, minimum distance to water was positively associated with CoVs (H). Note that these graphs represent the individual effects of landscape variables on raw data and are not corrected for the effect of other variables included in the GzLMMs.
(TIF)

S5 Fig Number of tubes and proportion of positive samples obtained per roosting category and season in *P*. *pipistrellus* (A) and *P*. *nathusii* (B). In the y-axis the number of faecal tubes collected and the proportion of positive tubes for CoVs is represented per sample origin (roosting size category) and seasonality. Note that for *P*. *nathusii* samples were not collected from large roosts as maternity colonies are not present in The Netherlands.
(ZIP)

S1 Appendix Detailed description of variables and model selection steps in the species *P*. *pipistrellus* and *P*. *nathusii*. (DOCX)

S2 Appendix R scripts associated with model selection in the species *P*. *pipistrellus* (a) and *P*. *nathusii* (b).
(ZIP)

S3 Appendix Raw data for model selection. (CSV)

S1 File (DOCX)
