# Supplementary figures and images for: Presence of coronaviruses in the common pipistrelle (P. pipistrellus) and Nathusius´ pipistrelle (P. nathusii) in relation to landscape composition
Source: PLoS One. 2023 Nov 29;18(11):e0293649. doi: 10.1371/journal.pone.0293649 (PMC10686486; doi:10.1371/journal.pone.0293649)

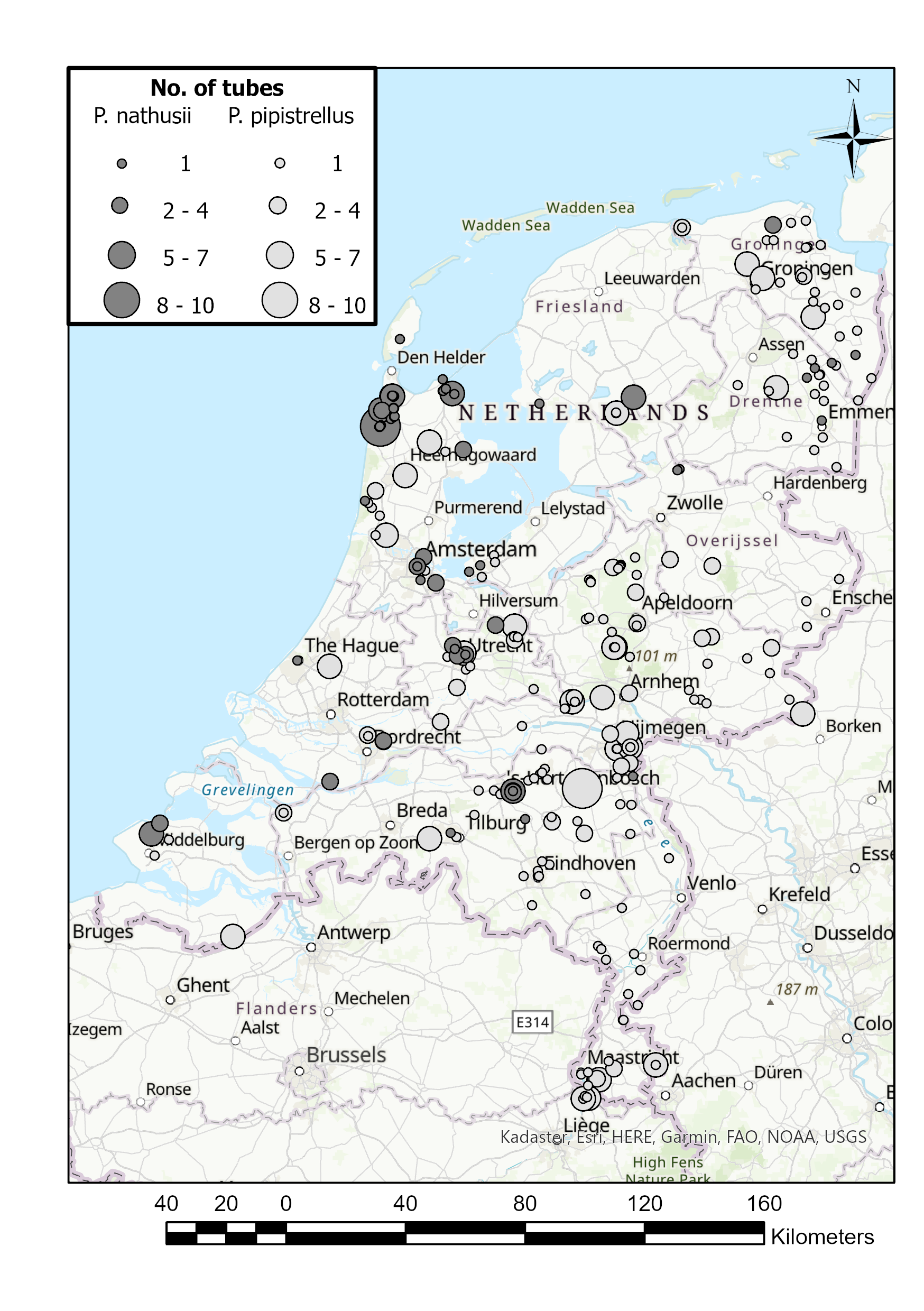

Supplement: S1 Fig — Source Basemap layer: World Topographic Map. Public domain Metadata. Credits attribution: Kadaster NL, Esri Inc, HERE, Garmin, FAO, NOAA, USGS. ArcGIS-Pro 2.5. https://services.arcgisonline.com/ArcGIS/rest/services/World_Topo_Map/MapServer Licensed under the Esri Master License Agreement. (TIF) [file pone.0293649.s001.tif]

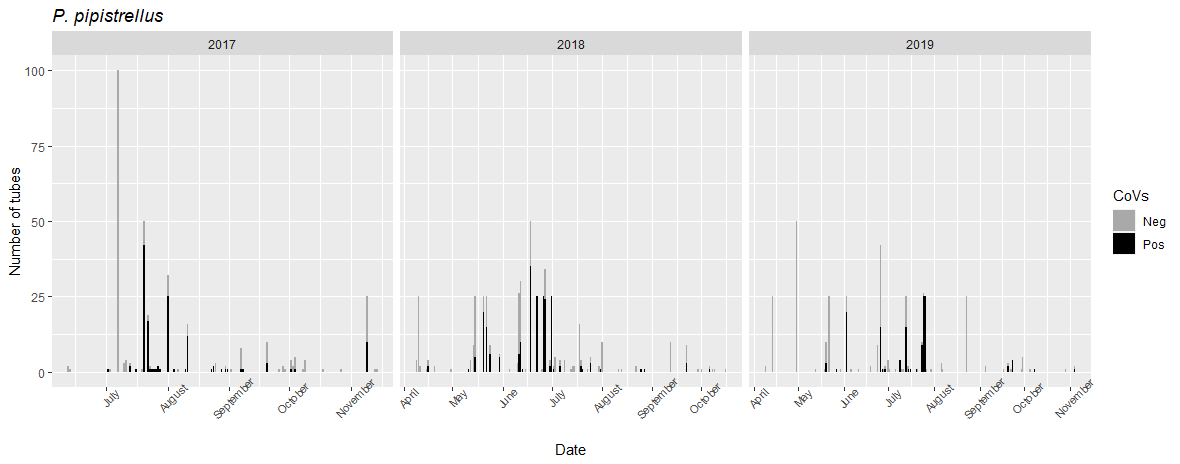

Supplement: S2 Fig — Temporal distribution of the number of tubes collected between 2017–2019, and the proportion of positive samples in P. pipistrellus (A) and P. nathusii (B). (ZIP) [file pone.0293649.s002.zip › S2 FigA.tiff]

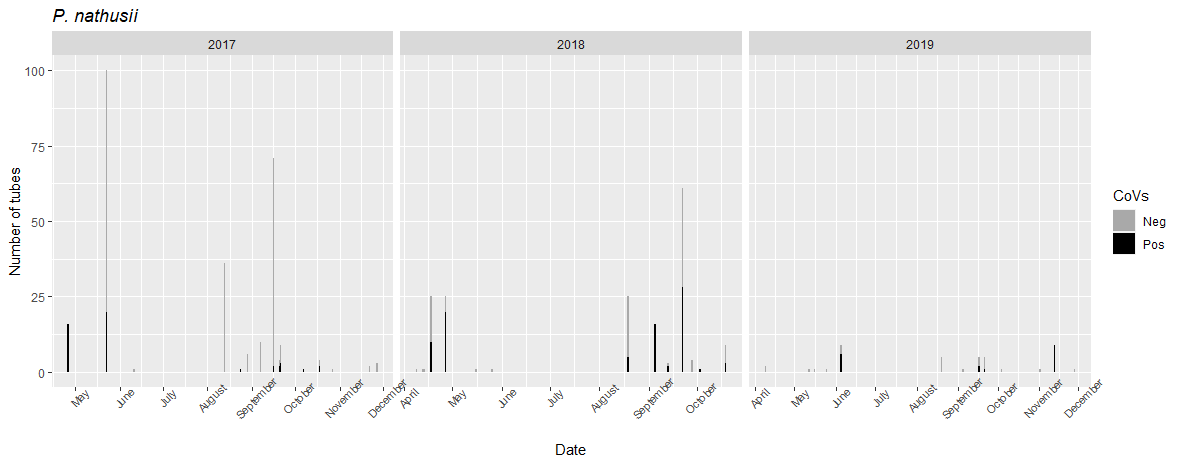

Supplement: S2 Fig — Temporal distribution of the number of tubes collected between 2017–2019, and the proportion of positive samples in P. pipistrellus (A) and P. nathusii (B). (ZIP) [file pone.0293649.s002.zip › S2 FigB.tiff]

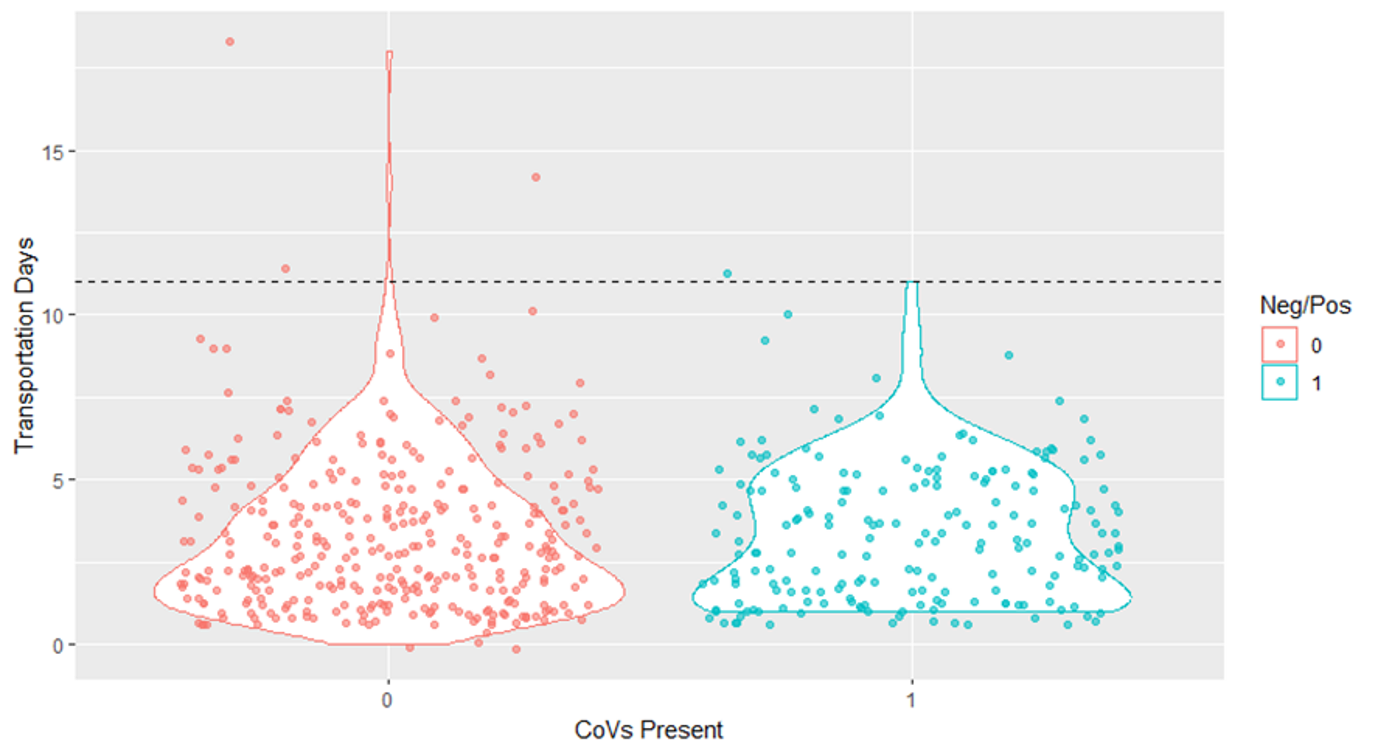

Supplement: S3 Fig — (TIF) [file pone.0293649.s003.tif]

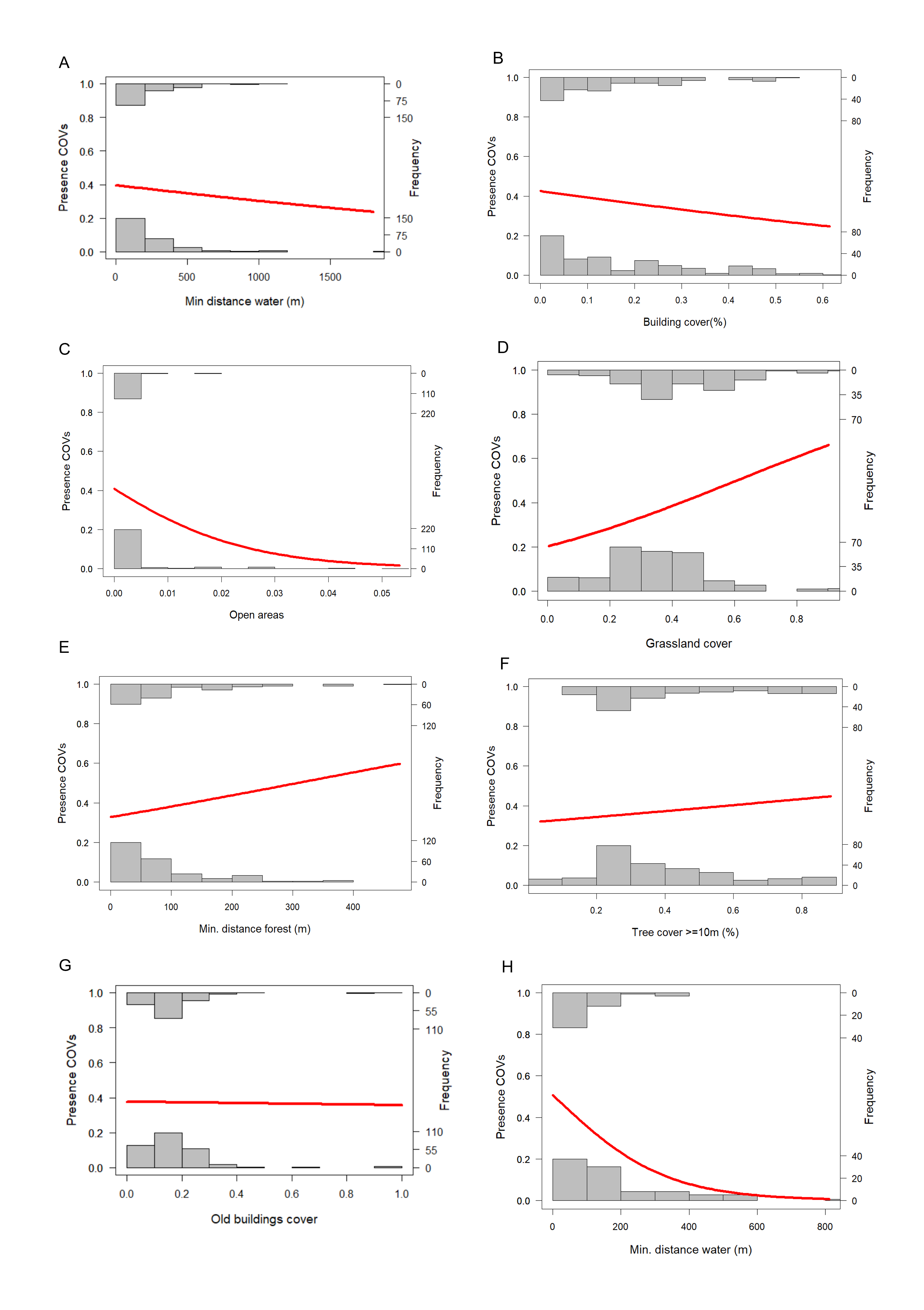

Supplement: S4 Fig — Correlations between landscape variables and presence of coronaviruses in P. pipistrellus (A-G) and P. nathusii (H). On the x-axis of each figure, histograms are displayed showing the distribution of each landscape variable across all faecal samples that are positive (above) and negative (below) for CoVs. On the y-axis the probability of coronavirus detection is represented. For P. pipistrelle min. distance to water (A), building cover (B), and open areas (C) were negatively correlated with the presence of CoVs, while grassland (D), minimum distance to forest (E), tree cover (> = 10m; F) and old buildings (G) had a positive correlation. In P. nathusii, minimum distance to water was positively associated with CoVs (H). Note that these graphs represent the individual effects of landscape variables on raw data and are not corrected for the effect of other variables included in the GzLMMs. (TIF) [file pone.0293649.s004.tif]

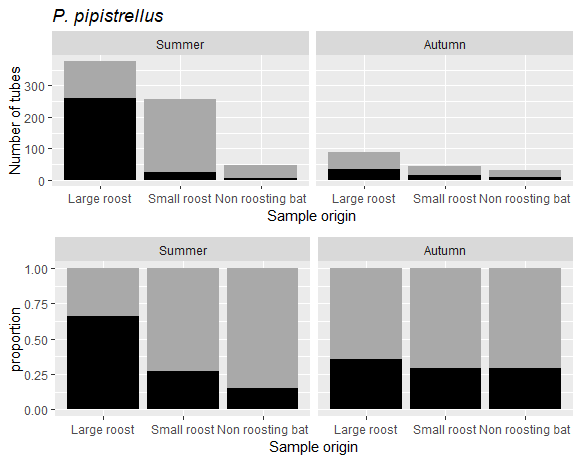

Supplement: S5 Fig — Number of tubes and proportion of positive samples obtained per roosting category and season in P. pipistrellus (A) and P. nathusii (B). In the y-axis the number of faecal tubes collected and the proportion of positive tubes for CoVs is represented per sample origin (roosting size category) and seasonality. Note that for P. nathusii samples were not collected from large roosts as maternity colonies are not present in The Netherlands. (ZIP) [file pone.0293649.s005.zip › S5 FigA.tiff]

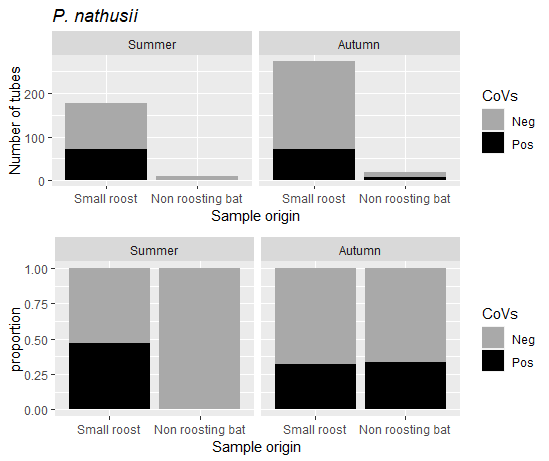

Supplement: S5 Fig — Number of tubes and proportion of positive samples obtained per roosting category and season in P. pipistrellus (A) and P. nathusii (B). In the y-axis the number of faecal tubes collected and the proportion of positive tubes for CoVs is represented per sample origin (roosting size category) and seasonality. Note that for P. nathusii samples were not collected from large roosts as maternity colonies are not present in The Netherlands. (ZIP) [file pone.0293649.s005.zip › S5 FigB.tiff]
