# Supplementary material for: Presence of coronaviruses in the common pipistrelle (P. pipistrellus) and Nathusius´ pipistrelle (P. nathusii) in relation to landscape composition
Source: PLoS One. 2023 Nov 29;18(11):e0293649. doi: 10.1371/journal.pone.0293649 (PMC10686486; doi:10.1371/journal.pone.0293649)

**Supplementary Information: Variables and Model selection (S1 Appendix)**


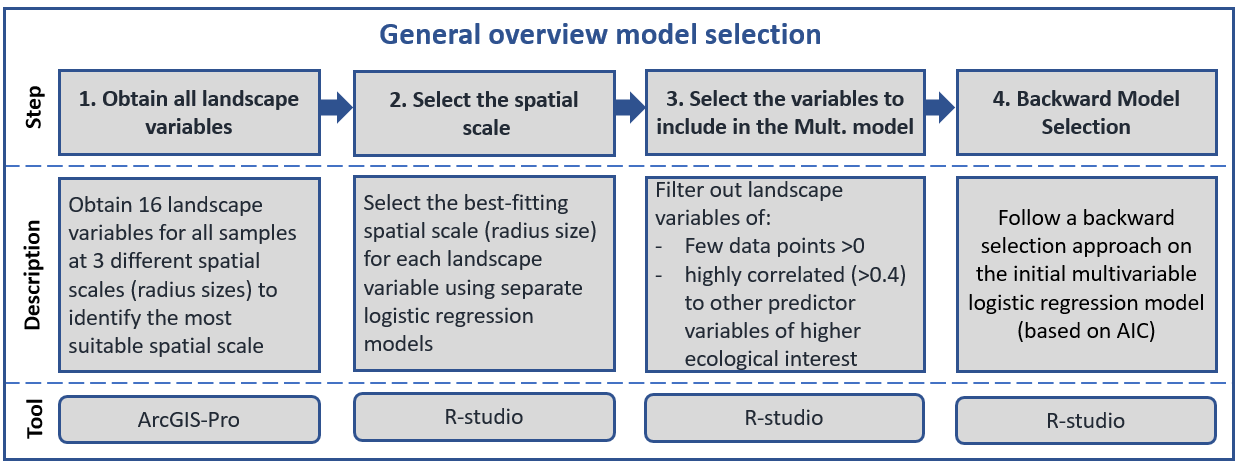


**S1A**: General description of the steps involved in the extraction of landscape variables, model construction, and model selection (backward selection) in both bat species: *P. pipistrellus* *and P. nathusii*.


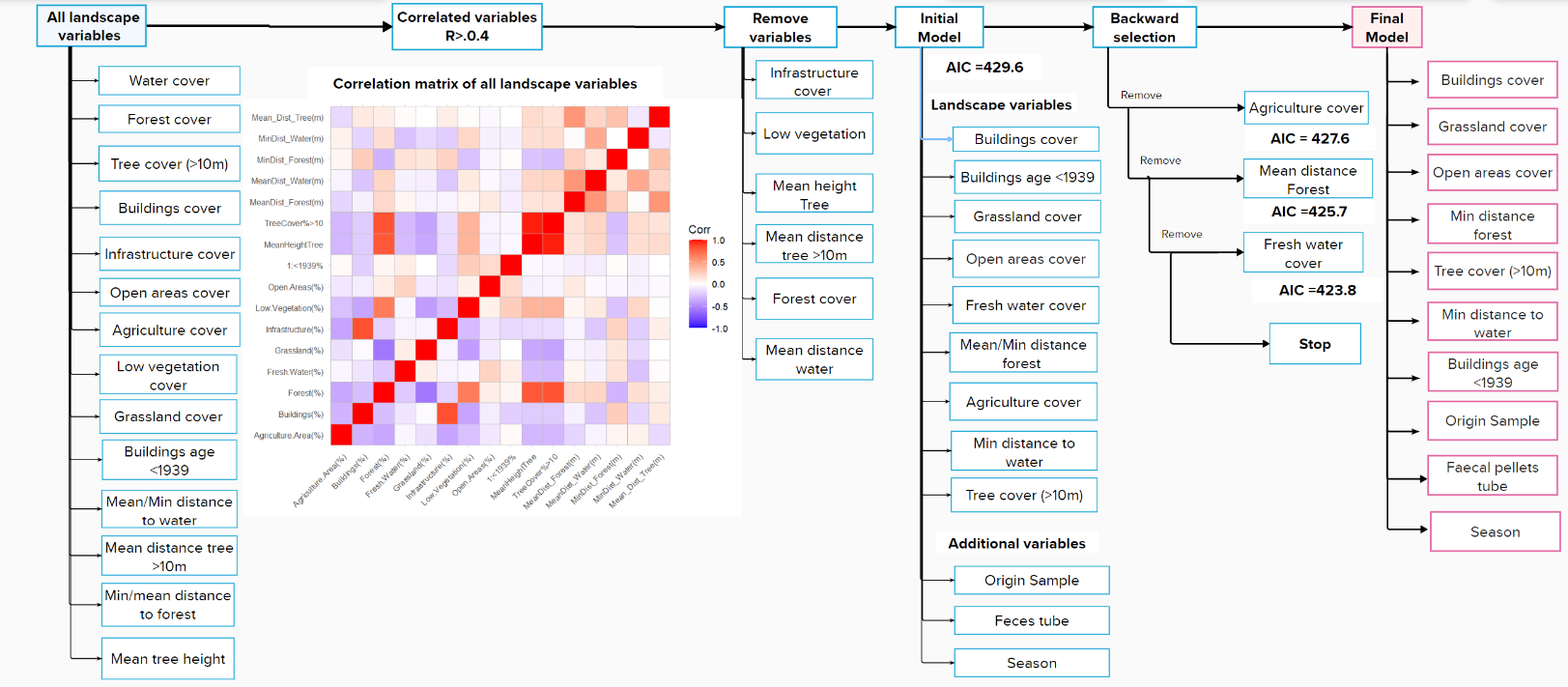


**S1B Figure:** **Detailed description of variable selection steps and model backward selection in *P. pipistrellus****.*  First, landscape variables were extracted from ArcGis. Next, highly correlated predictor variables of less theoretical relevance were excluded from the initial model using a conservative threshold of 0.4. All remaining landscape variables were used to form the initial model, using sample origin, faecal pellets per tube and seasonality as additional variables.


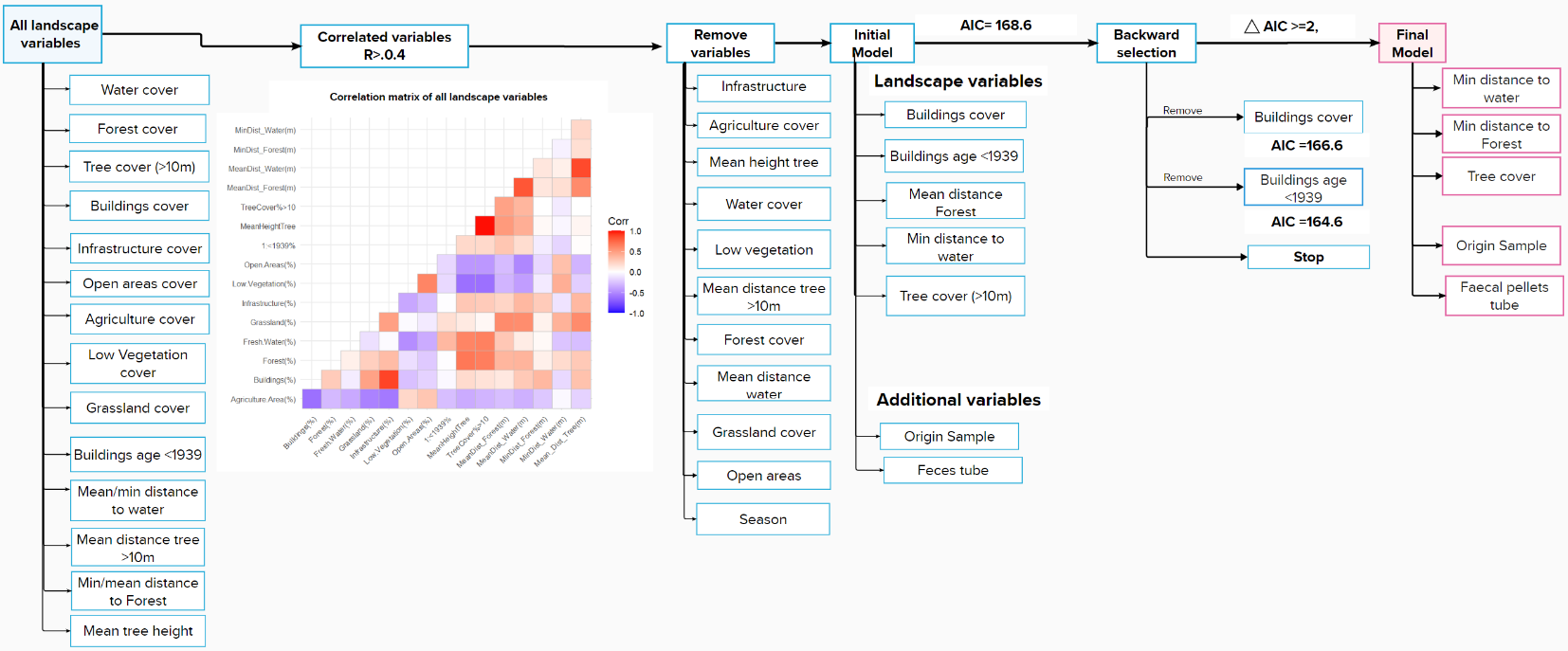


**S1C Figure: Detailed description of variable selection steps and model backward selection in *P. nathusii.*** First, landscape variables were extracted from ArcGis. Next, highly correlated predictor variables of less theoretical relevance were excluded from the initial model using a conservative threshold of 0.4. All remaining landscape variables were used to form the initial model, using sample origin and faecal pellets per tube as additional variables.


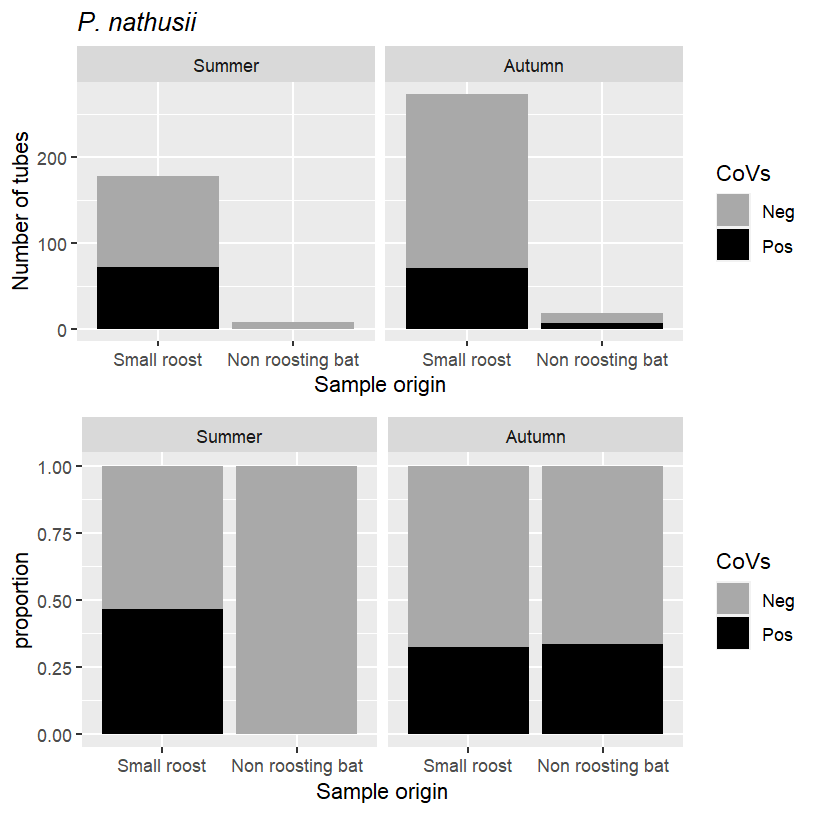

Supplement: S1 Appendix — (DOCX) [file pone.0293649.s006.docx]
