# Supplementary material for: Presence of coronaviruses in the common pipistrelle (P. pipistrellus) and Nathusius´ pipistrelle (P. nathusii) in relation to landscape composition
Source: PLoS One. 2023 Nov 29;18(11):e0293649. doi: 10.1371/journal.pone.0293649 (PMC10686486; doi:10.1371/journal.pone.0293649)
Supplement: S1 File — (DOCX) [file pone.0293649.s009.docx]

**S1 Table.** Size representation of spatial buffers. Buffer sizes per bat species are determined by home ranges reported in literature 1[29] , 2[38], 3[43], 4[60], 5[30]. Buffer 1= surrounding sample collection; buffer 2=core habitat; buffer 3= core habitat surrounding; buffer 4=maximum home range. Buffer dimensions are presented as radii.

| **Species** | **Buffer 1**  **Sample Surrounding** | **Buffer 2**  **Core habitat** | **Buffer 3**  **Core surrounding** | **Buffer 4**  **Max. range** |
| --- | --- | --- | --- | --- |
| *P. pipistrellus* | 200 m | 1.5 km^3,4,^ | 2 km*^1,3,4^* | 5 km*^1, 3^* |
| *P. nathusii* | 200 m | 2 km^2^ | 6.6 km^2,5^ | 12 km |

**S2 Table. LGN7 Database classification.** New classification for this study are defined in the column “study classification”.

| **Land-use Classification** | | |
| --- | --- | --- |
| **Major class (LGN7)** | **Minor classes  (LGN7)** | **Study classification** |
| Nature | Shrubs heaths, dunes with low vegetation (<1m) dunes with high vegetation (>1m), dune heather, heather, moderately grassed heather, greatly grazed heather, other swamp vegetation, reed  vegetation | Low/shrub vegetation cover (%) |
| Forest/  built-up area | Deciduous forest, coniferous forest, forest in  wetland, forest in moorland, forest in primarily built-up area, forest in secondary built-up area | Forest cover (%) |
| Fresh water | Fresh water | Freshwater cover (%) |
| Salt water  /Nature | Salt water, salt marshes | Saltwater cover (%) |
| Infrastructure | Main roads and railways | Infrastructure cover (%) |
| Built-up area | Buildings in rural areas, buildings in primarily built- up area, buildings in secondary built-up area | Buildings cover (%) |
| Nature/ built-up areas /agriculture areas | Open sand/ river sand, bare land in built-up rural areas, open sand in coastal area, greenhouse horticulture | Open areas cover (%) |
| Nature/ Built-up areas | Agricultural grass, natural grassland, grass in secondary built-up area, grass in primarily built-up area | Grassland cover (%) |
| Agriculture areas | Maize, potatoes, beets, grains, cereal, fruits, orchard, bulbs, other agricultural crops, nurseries | Agriculture cover (%) |

**S3 Table. Description of the landscape variables used for the spatial analysis**. Each landscape variable has a corresponding name and data source. Landscape variables are categorised into land use variables and landscape elements.

| **Landscape variables** | **Data source** | |
| --- | --- | --- |
| 1. Agriculture cover (%) |  | LGN 7 |
| 2. Buildings cover (%) |  | LGN 7 |
| 3. Forest cover (%) |  | LGN 7 |
| 4. Freshwater cover (%) |  | LGN 7 |
| 5. Grassland cover (%) |  | LGN 7 |
| 6. Open areas cover (%) |  | LGN 7 |
| 7. Infrastructure cover (%) |  | LGN 7 |
| 8. Low vegetation cover (%) |  | LGN 7 |
| **Landscape elements** |  |  |
| 9. Old buildings cover (%): buildings constructed before 1939 |  | BAG-2018 |
| 10. Tree cover (≥10 m) (%) |  | Boombasis data |
| 11. Mean tree height (m) |  | Boombasis data |
| 12 Mean distance to border of trees ≥10m |  | Boombasis data |
| 13-14. Mean/min. distance to border of forest (m) |  | LGN 7 |
| 15-16. Mean/min. distance to border of fresh water (m) | | LGN 7 |

**S4 Table. Description of the inclusive R^2^ for all predictor variables with the corresponding confidence intervals.**

| **Predictor** | **Inclusive R^2^** | **95% CI** |
| --- | --- | --- |
| ***P. pipistrellus*** | | |
| Old_Buildings | 0.0008 | 0, 0.0312 |
| Buildings | 0.0394 | 0.0012, 0.0919 |
| Tree_cover | 0.0163 | 0.000, 0.0743 |
| Grassland_cover | 0.0333 | 0.0009, 0.1342 |
| Open_areas | 0.0545 | 0.004, 0.2573 |
| Min_dist_forest | 0.005 | 0 , 0.0426 |
| Min_dist_water | 0.0013 | 0, 0.0435 |
| Sample_sourceIndividual | 0.0439 | 0.0008, 0.1133 |
| Sample_sourceUnknown | 0.0001 | 0, 0.0224 |
| Faeces_Tube | 0.0251 | 0.0007, 0.094 |
| SeasonSummer | 0.0206 | 0.0001, 0.0684 |
| ***P. nathusii*** | | |
| Tree_cover | 0.0127 | 0.0002, 0.0762 |
| Min_dist_water | 0.1666 | 0.0333, 0.4391 |
| Min dist forest | 0.0076 | 0.000, 0.1873 |
| Sample_sourceIndividual | 0.0192 | 0, 0.1086 |
| Faeces_Tube | 0.0188 | 0.0001, 0.0863 |

**S5 Table. Standardized odd ratio values for all predictor variables with the corresponding confidence intervals.**

| **Predictor** | **Odd ratio** | **95% CI** |
| --- | --- | --- |
| ***P. pipistrellus*** | | |
| Old_Buildings | 1.19 | 0.67, 2.11 |
| Buildings | 0.45 | 0.22, 0.90 |
| Tree_cover | 1.96 | 0.86, 4.44 |
| Grassland_cover | 1.94 | 0.96, 3.93 |
| Open_areas | 0.43 | 0.17, 1.06 |
| Min._dist_forest | 1.95 | 1.01, 3.76 |
| Min._dist_water | 0.75 | 0.42, 1.33 |
| Sample_sourceIndividual | 0.12 | 0.02, 0.58 |
| Sample_source Unknown | 0.50 | 0.12, 2.01 |
| Faeces_Tube | 1.55 | 0.94, 2.55 |
| SeasonSummer | 1.78 | 0.53, 5.9 |
| ***P. nathusii*** | | |
| Tree_cover | 1.40 | 0.89, 2.23 |
| Min._dist_water | 0.36 | 0.18, 0.73 |
| Min._dist_forest | 0.85 | 0.51, 1.40 |
| Sample_source Non roosting bat | 0.28 | 0.06, 1.24 |
| Faeces_Tube | 1.61 | 1.00, 2.61 |
